# Divergent skeletal muscle mitochondrial phenotype between male and female patients with chronic heart failure

**DOI:** 10.1002/jcsm.12488

**Published:** 2019-08-20

**Authors:** Jack O. Garnham, Lee D. Roberts, Talia Caspi, Moza M. Al‐Owais, Max Bullock, Peter P. Swoboda, Aaron Koshy, John Gierula, Maria F. Paton, Richard M. Cubbon, Mark T. Kearney, T. Scott Bowen, Klaus K. Witte

**Affiliations:** ^1^ Leeds Institute of Cardiovascular and Metabolic Medicine University of Leeds Leeds UK; ^2^ School of Biomedical Sciences, Faculty of Biological Sciences University of Leeds Leeds UK

**Keywords:** HFrEF, Mitochondrial dysfunction, OPA1, Exercise intolerance, Sex

## Abstract

**Background:**

Previous studies in heart failure with reduced ejection fraction (HFrEF) suggest that skeletal muscle mitochondrial impairments are associated with exercise intolerance in men. However, the nature of this relationship in female patients remains to be elucidated. This study aimed to determine the relationship between skeletal muscle mitochondrial impairments and exercise intolerance in male and female patients with HFrEF.

**Methods:**

Mitochondrial respiration, enzyme activity, and gene expression were examined in *pectoralis major* biopsies from age‐matched male (*n* = 45) and female (*n* = 11) patients with HFrEF and healthy‐matched male (*n* = 24) and female (*n* = 11) controls. Mitochondrial variables were compared between sex and related to peak exercise capacity.

**Results:**

Compared with sex‐matched controls, complex I mitochondrial oxygen flux was 17% (*P* = 0.030) and 29% (*P* = 0.013) lower in male and female patients with HFrEF, respectively, which correlated to exercise capacity (*r* = 0.71; *P* > 0.0001). Female HFrEF patients had a 32% (*P* = 0.023) lower mitochondrial content compared with controls. However, after adjusting for mitochondrial content, male patients demonstrated lower complex I function by 15% (*P* = 0.030). Expression of key mitochondrial genes regulating organelle dynamics and maintenance (i.e. optic atrophy 1, peroxisome proliferator‐activated receptor γ coactivator‐1α, NADH:ubiquinone oxidoreductase core subunit S1/S3, and superoxide dismutase 2) were selectively lower in female HFrEF patients.

**Conclusions:**

These data provide novel evidence that HFrEF induces divergent sex‐specific mitochondrial phenotypes in skeletal muscle that predispose towards exercise intolerance, impacting mitochondrial ‘quantity' in female patients and mitochondrial ‘quality' in male patients. Therapeutic strategies to improve exercise tolerance in HFrEF should consider targeting sex‐specific mitochondrial abnormalities in skeletal muscle.

## 1 Introduction

Patients with heart failure with reduced ejection fraction (HFrEF) have a complex pathophysiology, which, although underpinned by left ventricular systolic dysfunction, is a multifaceted whole‐body syndrome characterized by exercise intolerance. The prevalence of HFrEF is similar between men and women, yet despite presenting with lower exercise capacity [i.e. peak pulmonary oxygen uptake (V̇O_2peak_)], women show better clinical outcomes both in the absence and in the presence of an exercise intervention when compared with male counterparts^1^, ^2^. The underlying sex‐specific mechanism(s) for this trend remain poorly resolved and seem independent of cardiac function.^3^, ^4^ Exercise intolerance in patients with HFrEF is poorly correlated with cardiac dysfunction^5^, ^6^, ^7^ and is accepted that peripheral impairments, including skeletal muscle abnormalities, play a large role in exercise intolerance in HFrEF.^8^ Mitochondria represent the key site for cellular energy production, and skeletal muscle mitochondrial impairments in HFrEF patients could predispose towards exercise intolerance.

Whether HFrEF induces sex‐specific mitochondrial decrements that contribute to the observed clinical differences between men and women with HFrEF remains poorly explored.^9^, ^10^ However, recent advances in the mitochondrial field have given rise to putative mechanisms that could form the basis of any sex‐specific mitochondrial impairments induced by HFrEF. Skeletal muscle oxidative capacity is determined by both mitochondrial ‘quantity' (mitochondrial content) and ‘quality' (intrinsic function normalized to mitochondrial mass). Mitochondrial quality and morphology are dependent on the effective functioning/expression of mitochondrial respiratory chain complexes, which are largely orchestrated by a set of regulatory proteins that act to maintain mitochondrial quality control in a process termed mitochondrial dynamics. Mitochondrial dynamics modulate mitochondrial shape, size, and integrity, which are regulated by fusion proteins [e.g. optic atrophy 1 (OPA1)] and fission proteins [e.g. mitochondrial fission 1 (FIS1)] localized to the inner and outer mitochondrial membranes, respectively.^11^ One major orchestrator of these mitochondrial processes is the well‐known transcriptional coactivator peroxisome proliferator‐activated receptor γ coactivator‐1α (PGC‐1α), which is considered as the master regulator of mitochondrial biogenesis.^12^

In the current study, therefore, we assessed skeletal muscle biopsies from a large cohort of both male and female patients with HFrEF compared with sex‐matched controls. We evaluated whether mitochondrial quality, quantity, respiratory chain complexes, and dynamics were impacted by HFrEF in a sex‐specific manner and their relationship to whole‐body exercise intolerance.

## 2 Methods

### 2.1 Participants

All participant characteristics are presented in *Table* 1. We approached consecutive patients with HFrEF of >3 months duration, symptoms corresponding to New York Heart Association (NYHA) functional class ≥ I and a documented left ventricular ejection fraction <50% (following current European Society of Cardiology guidelines^13^) who were planned for primary electronic cardiac implantable device procedures. All were indicated for device therapy with either an implantable cardioverter defibrillator or cardiac resynchronization therapy according to current indications. Pacemaker requirement was sick sinus syndrome, heart block, or cardiac arrest (implantable cardioverter defibrillator patients). All HFrEF patients undertook a peak symptom‐limited CPX test to volitional exhaustion on a cycle ergometer for determination of pulmonary gas exchange (V̇O_2_ and V̇CO_2_) and ventilation (V̇_E_).^14^ Control subjects were consecutive patients listed for device implantation of a permanent pacemaker without symptoms of heart failure. Potential participants were excluded if they were unable to provide informed consent due to cognitive dysfunction or had previously been diagnosed with other potentially confounding comorbidities, such as other cardiovascular conditions, chronic obstructive pulmonary disease, or cancer. All patients provided written informed consent, and all procedures were conducted in accordance with the Declaration of Helsinki after receiving local institute ethical approval (11/YH/0291).

**Table 1:** Physical, clinical, and treatment characteristics of patients

	CON			HFrEF		
	Male (*n* = 24)	Female (*n* = 11)	*P* value	Male (*n* = 45)	Female (*n* = 13)	*P* value
Age (years)	70.1 ± 2.6	74.1 ± 3.1	0.33	71.8 ± 1.8	69.9 ± 2.1	0.45
BMI (kg·m^−2^)	27.4 ± 0.5	29.6 ± 2.7	0.46	28.1 ± 0.9	27.2 ± 1.8	0.67
V̇O_2peak_ (mL·kg^−1^·min^−1^)	—	—	—	15.1 ± 0.8	11.6 ± 0.7	0.07
V̇_E_/V̇CO_2_ slope	—	—	—	35.6 ± 1.7	37.9 ± 3.0	0.56
NYHA functional class [% (*n*)]	—	—		—	—	—
I	—	—		13.3 (6)	7.7 (1)	0.18
II	—	—		60.0 (27)	38.5 (5)	
III	—	—		26.7 (12)	53.8 (7)	
Ischaemic/DCM aetiology [% (*n*)]	—	—	—	58/42 (26/19)	39/61 (5/8)	0.22
AF [% (*n*)]	—	—	—	37.8 (17)	15.4 (2)	0.13
CABG [% (*n*)]	—	—	—	24.4 (11)	7.7 (1)	0.19
Hypertension [% (*n*)]	—	—	—	33.3 (15)	30.8 (4)	0.86
LVEF (%)	—	—	—	24.6 ± 1.5	25.4 ± 3.4	0.83
LVIDd (mm)	—	—	—	59.3 ± 1.3	56.5 ± 1.5	0.18
HbA1c (%)	5.90 ± 0.12	5.78 ± 0.13	0.53	6.01 ± 0.11	5.90 ± 0.09	0.63
Haemoglobin (g·L^−1^)	138.0 ± 3.4	124.9 ± 6.2	0.08	140.5 ± 2.0	131.6 ± 3.5	0.04
Creatinine (μmol·mL^−1^)	83.2 ± 3.1	75.9 ± 4.5	0.19	99.6 ± 6.0	82.8 ± 7.8	0.10
Pharmacological treatments	—	—	—	—	—	—
Beta‐blocker use [% (*n*)]	—	—	—	97.8 (44)	84.6 (11)	0.06
ACEi or ARB use [% (*n*)]	—	—	—	86.7 (39)	69.2 (9)	0.14
MRA use [% (*n*)]	—	—	—	53.3 (24)	46.2 (6)	0.64
ACEi or ARB with beta‐blocker and MRA [% (*n*)]	—	—	—	46.7 (21)	38.5 (5)	0.52
Loop diuretic use [% (*n*)]	—	—	—	87.1 (27)	30.8 (4)	0.06
Furosemide equivalent dose (mg)	—	—	—	45.9 ± 4.2	55.0 ± 15.0	0.53
Statin use [% (*n*)]	—	—	—	60.0 (27)	69.2 (9)	0.54
Statin dose (mg)	—	—	—	48.9 ± 4.8	24.4 ± 4.1	<0.01
Aspirin use [% (*n*)]	—	—	—	37.8 (17)	53.8 (7)	0.30
Digoxin use [% (*n*)]	—	—	—	11.1 (5)	7.7 (1)	0.72
Device therapy	—	—	—	—	—	—
Pacemaker	91.7 (22)	90.9 (10)	0.94	2.2 (1)	0 (0)	0.59
ICD [% (*n*)]	8.3 (2)	9.1 (1)	0.94	24.4 (11)	15.4 (2)	0.49
CRT [% (*n*)]	—	—	—	37.8 (17)	61.5 (8)	0.13
CRT‐ICD [% (*n*)]	—	—	—	35.6 (16)	23.1 (3)	0.40

Data are presented as mean ± SEM or % (*n*). Continuous variables were compared using unpaired Student's *t*‐tests. Categorical variables were compared using chi‐squared tests (or Fisher's exact test were appropriate). ACEi, angiotensin‐converting enzyme inhibitor; AF, atrial fibrillation; ARB, angiotensin receptor blocker; BMI, body mass index; CABG, coronary artery bypass graft; CRT, cardiac resynchronization therapy; DCM, dilated cardiomyopathy; HbA1c, glycated haemoglobin; ICD, implantable cardioverter‐defibrillator; LVEF, left ventricular ejection fraction; LVIDd, left ventricular internal diameter at diastole; MRA, mineralocorticoid antagonist; NYHA, New York Heart Association; V̇O_2peak_, peak pulmonary oxygen uptake; V̇_E_/V̇CO_2_, ratio of ventilation to carbon dioxide production.

### 2.2 Skeletal muscle biopsy

Skeletal muscle biopsies of *pectoralis major* (~100 mg) with a mixed fibre type composition (~35% type I, ~40% type IIA, and ~25% type IIX) were obtained during otherwise routinely performed device implantation procedures. The biopsy was taken within a maximum of 3 months of re‐examination of disease status at the time of decision to implant a device. There were no complications or adverse events with this procedure. One piece of muscle sample was immediately place in 1 mL of ice‐cold specialized preservation solution,^15^ while another portion was snap frozen in liquid nitrogen and stored at −80°C.

### 2.3 Mitochondrial function and content

Mitochondrial respiration was assessed *in situ* from saponin‐permeabilized skeletal muscle fibres using high‐resolution respirometry (Oxygraph‐2K; Oroboros Instruments, Innsbruck, Austria), as described by Wüst *et al*.^16^ briefly in the following order: (i) complex I leak respiration was determined by addition of glutamate (10 mM), malate (0.5 mM), and pyruvate (5 mM) (i.e. a measure of proton leak under non‐phosphorylating conditions); (ii) adenosine diphosphate (2.5 mM) added to provide a measure of complex I oxidative phosphorylation (OXPHOS); (iii) outer mitochondrial membrane integrity determined by addition of 10 μM cytochrome *c*; (iv) succinate (10 mM) as a complex II substrate provided complex I + II OXPHOS; (v) 5 μM FCCP for maximal uncoupled complex I + II respiration; (vi) complex I inhibitor rotenone (0.25 μM) provided uncoupled complex II respiration; and (vii) 2.5 μM antimycin A as a complex III inhibitor for residual oxygen consumption (ROX) to calculate non‐mitochondrial (background) respiration, which was then subtracted from the other data. Mitochondrial content was first determined within the respirometer by the addition of a complex IV activity assay,^17^ by the addition of 0.5 mM N,N,N′,N′‐tetramethyl‐p‐phenylenediamine dihydrochloride (TMPD) as an artificial electron donor to complex IV in combination with 2 mM ascorbate to maintain TMPD in a reduced state,^18^ with absolute mitochondrial respiration states thereafter normalized to complex IV activity to provide an index of mitochondrial intrinsic function (i.e. quality). Citrate synthase activity was also measured spectrophotometrically at 30°C in a plate reader at 412 nm in muscle homogenate as a secondary marker of mitochondrial content and specific enzyme activity was calculated.^19^ Complex IV activity and specific enzyme activity of citrate synthase were expressed as a fold change relative to controls.

### 2.4 Gene and protein expression analyses

RNA was extracted and purified from ~30 to 40 mg snap‐frozen muscle tissue using the RNeasy® Fibrous Tissue Mini Kit (Qiagen, Hilden, Germany). RNA concentrations (ng·μL^−1^) were quantified and reverse transcribed to cDNA and mRNA expression was determined using real‐time quantitative PCR with SYBR® Green ROX™ quantitative PCR Mastermix (QIAGEN, Hilden, Germany) and a quantitative PCR system (Applied Biosystems Prism 7900HT, Foster City, CA). Primers were purchased from Qiagen including PGC‐1α, superoxide dismutase 2 (SOD2), FIS1, OPA1, NADH:ubiquinone oxidoreductase core subunit S1 (NDUFS1), and NADH:ubiquinone oxidoreductase core subunit S3 (NDUFS3). Expression levels were normalized to an endogenous control, beta‐actin, using the ^Δ‐Δ‐^C_T_ method^20^ and then expressed relative to controls. All real‐time quantitative PCR primers used in this study are commercially available (Qiagen Ltd) and are as follows: PPARGC1A, detected transcript NM_013261 (6318 bp) (PPH00461F); OPA1, detected transcript NM_015560 (6345 bp) (PPH12084A‐200); FIS1, detected transcript NM_016068 (785 bp) PPH19947A‐200; SOD2, detected transcript NM_000636 (1593 bp) PPH01716B‐200; NDUFS1, detected transcript NM_001199981 (3365 bp) (PPH19871A‐200); and NDUFS3, detected transcript NM_004551 (971 bp) PPH05975A‐200. For protein expression, homogenized muscle samples underwent sodium dodecyl sulfate (SDS)‐polyacrylamide gel electrophoresis, membrane transfer, and were incubated overnight at 4°C with a primary antibody against PGC‐1α (1/200; GeneTex) and subsequently with a horseradish peroxidase‐conjugated secondary antibody before visualization by enzymatic chemiluminescence and quantification using densitometry, as previously described.^19^ Blots were normalized to the loading control GAPDH (1/5000, Abcam).

### 2.5 Statistical analyses

Homogeneity of variance and normal distribution was first confirmed, then one‐way analysis of variance was used to compare all four patient cohorts. Differences in V̇O_2peak_, NYHA functional class, echocardiography, and clinical and treatment variables between HFrEF men and HFrEF women were determined using unpaired Student's *t*‐tests. Categorical variables were compared using chi‐squared tests (or Fisher's exact test were appropriate). For all mitochondrial function and content data as well as gene expression analyses, Student's *t*‐tests were employed for within‐sex comparisons. Pearson correlations were included to examine relationships between measures of mitochondrial content and function with V̇O_2peak_. Statistical significance was accepted as *P* < 0.05, and all data were analysed using commercial software (SPSS for Windows Version 25.0; IBM Corporation, Armonk, NY).

## 3 Results

### 3.1 Patient characteristics

Characteristics for all patients are presented in *Table* 1. The four cohorts showed no differences between age and body mass index. Patients with HFrEF had symptoms of exercise intolerance and left ventricular systolic dysfunction, with no significant differences between sexes in terms of NYHA functional class, aetiology, glycated haemoglobin, additional comorbidities, V̇O_2peak_, and cardiac function. Pharmacological treatments and device therapies were also not different between men and women in the HFrEF group.

### 3.2 Mitochondrial respiration and content

**Figure 1:**
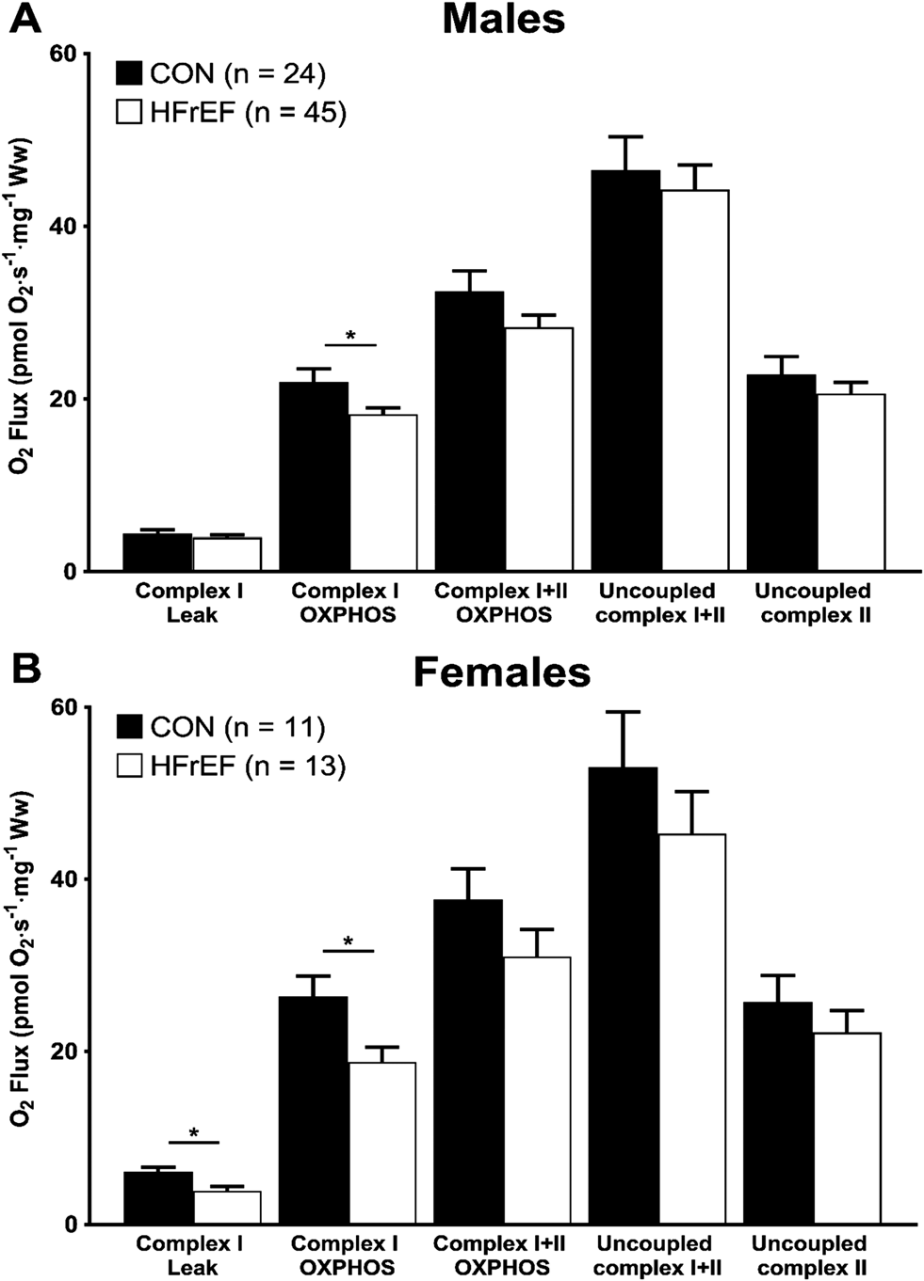
Skeletal muscle mitochondrial O_2_ flux across all respiratory states in male and female patients. Mitochondrial oxygen flux with complex I substrates is lower in both male (A) and female (B) patients with HFrEF compared with age‐matched and sex‐matched controls. Data are mean ± SEM. *P < 0.05 using unpaired Student's *t*‐tests. HFrEF, heart failure with reduced ejection fraction; OXPHOS, oxidative phosphorylation.

**Figure 2:**
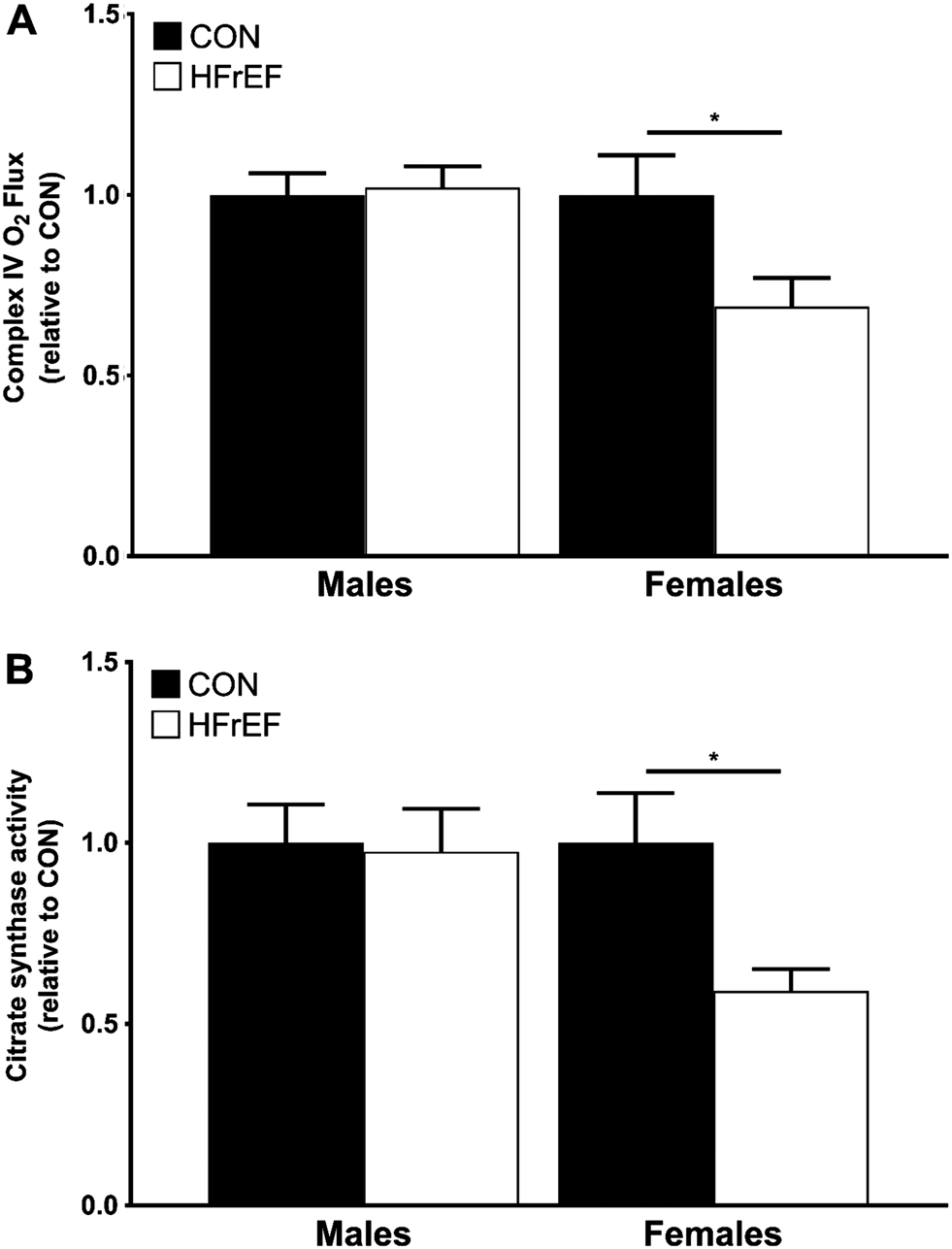
Relative measures of complex IV and citrate synthase activities as markers of mitochondrial content. Mitochondrial content determined from complex IV activity (A) was lower in women with HFrEF (*n* = 13) compared with female controls (*n* = 11) but did not differ between men with (*n* = 45) or without (*n* = 24) HFrEF. Similarly, citrate synthase activity (B) was also lower in women with HFrEF (*n* = 10) compared with women (*n* = 8) but men with HFrEF (*n* = 9) did not differ to sex‐matched controls (*n* = 8). Data are mean ± SEM. *P < 0.05 using unpaired Student's *t*‐tests. HFrEF, heart failure with reduced ejection fraction.

**Figure 3:**
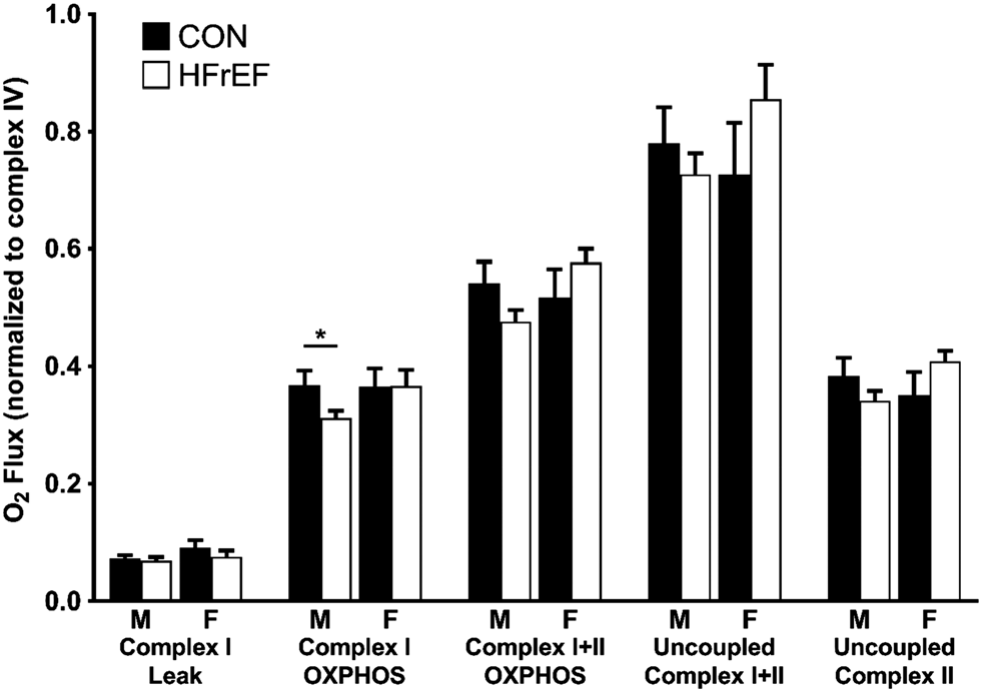
Skeletal muscle mitochondrial O_2_ flux across all respiratory states after adjusting the data to complex IV activity. After adjusting for mitochondrial content, complex I OXPHOS O_2_ flux was only lower in male (M) patients with HFrEF (*n* = 45) compared with male controls (*n* = 24). There were no differences between female (F) patients with HFrEF (*n* = 13) and female controls (*n* = 11). Data are mean ± SEM. *P < 0.05 using unpaired Student's *t*‐tests. HFrEF, heart failure with reduced ejection fraction; OXPHOS, oxidative phosphorylation.

Compared with sex‐matched control subjects, mitochondrial respiration with substrates supporting complex I OXPHOS was lower by 17.2% (*P* = 0.030; *Figure* 1A) in men with HFrEF and 28.9% (*P* = 0.013; *Figure* 1B) in women with HFrEF. Oxygen flux with complex I leak substrates was also 35.2% (*P* = 0.010; *Figure* 1B) lower in women with HFrEF compared with women in the control group. Mitochondrial content, as assessed by complex IV activity, was 31.5% (*P* = 0.023; *Figure* 2A) lower in women with HFrEF compared with female controls, whereas complex IV activity was not different in the male patients with HFrEF compared with controls (*P* > 0.05; *Figure* 2A). We also confirmed this by measuring citrate synthase enzyme activity as a secondary, independent measure of mitochondrial content (*Figure* 2B). This measure demonstrated that mitochondrial content was 40.9% lower in female patients with HFrEF (*P* = 0.011; *Figure* 2B) while there were no differences between the two male cohorts (*P* > 0.05; *Figure* 2B). There was a positive and significant correlation between complex IV activity and citrate synthase activity (*r* = 0.420; *P* = 0.012), thereby validating these two independent measures of mitochondrial content. When the mitochondrial respiration was normalized to complex IV activity (and therefore mitochondrial content), there were no differences between women with and without HFrEF (all *P* > 0.05; *Figure* 3); however, complex I OXPHOS was still 15.4% (*P* = 0.030; *Figure* 3) lower in men with HFrEF than male controls. Overall, these data show women with HFrEF have lower mitochondrial content whereas men with HFrEF have complex I‐specific impaired mitochondrial quality. The respiratory control ratio (ratio of complex I OXPHOS to leak respiration) was not different between control and HFrEF groups in both men (5.30 ± 0.67 vs. 5.25 ± 0.62; *P* > 0.05) and women (4.57 ± 0.46 vs. 5.06 ± 0.56; *P* > 0.05). While flux control ratio (ratio of individual complex respiration to maximal uncoupled respiration) was not different between male groups for complex I (0.51 ± 0.04 vs. 0.45 ± 0.02, *P* > 0.05), it was lower in the women with CHF relative to the controls (0.55 ± 0.05 vs. 0.44 ± 0.03; *P* = 0.045). No other differences in the flux control ratio across the respiratory states was otherwise noted.

Additional between‐group comparisons for mitochondrial measures between all cohorts revealed further differences, including female controls having higher values than CHF men for mitochondrial respiration during leak respiration (*P* = 0.02), complex I OXPHOS (*P* = 0.001), and complex I + II OXPHOS (*P* = 0.03) (*Figure* 1). No further differences between all cohorts were detected in terms of mitochondrial density (*Figure* 2), normalized mitochondrial function (*Figure* 3), or in the respiratory or flux control ratios (all *P* > 0.05).

### 3.3 Gene and protein expression

**Figure 4:**
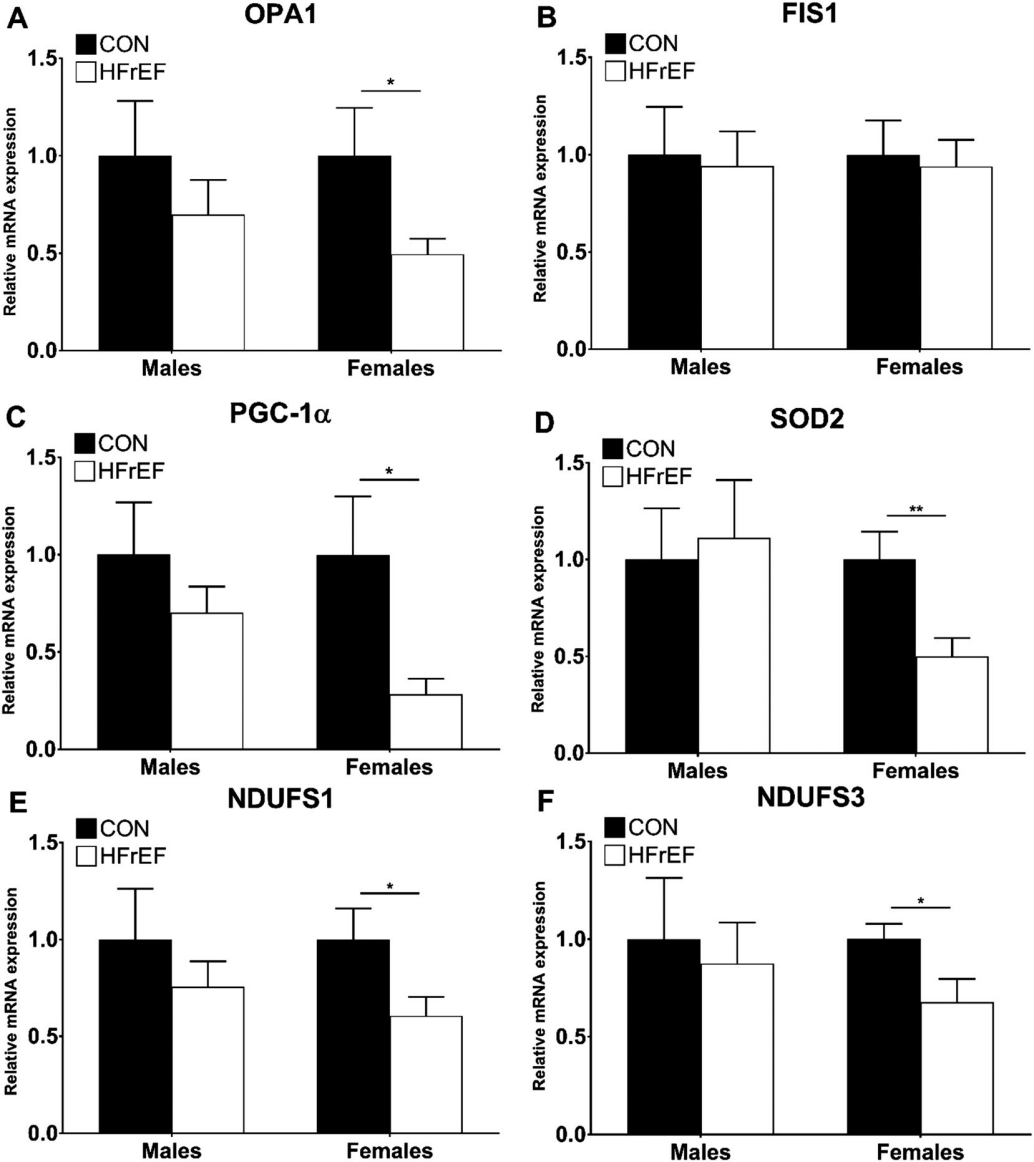
Relative mRNA expression levels of several key mitochondrial gene transcripts. Compared with controls, female patients with HFrEF have lower relative mRNA expression for OPA1 (A), PGC‐1α (C), SOD2 (D), NDUFS1 (E), and NDUFS3 (F) (*n* = 10 per group for each sex). *P < 0.05 using unpaired Student's *t*‐tests. ^**^ *P* < 0.01 using unpaired Student's *t*‐tests. FIS1, mitochondrial fission 1; OPA1, optic atrophy 1; NDUFS1, NADH:ubiquinone oxidoreductase core subunit S1; NDUFS3, NADH:ubiquinone oxidoreductase core subunit S3; PGC‐1α, peroxisome proliferator‐activated receptor γ coactivator‐1α; SOD2, superoxide dismutase 2.

**Figure 5:**
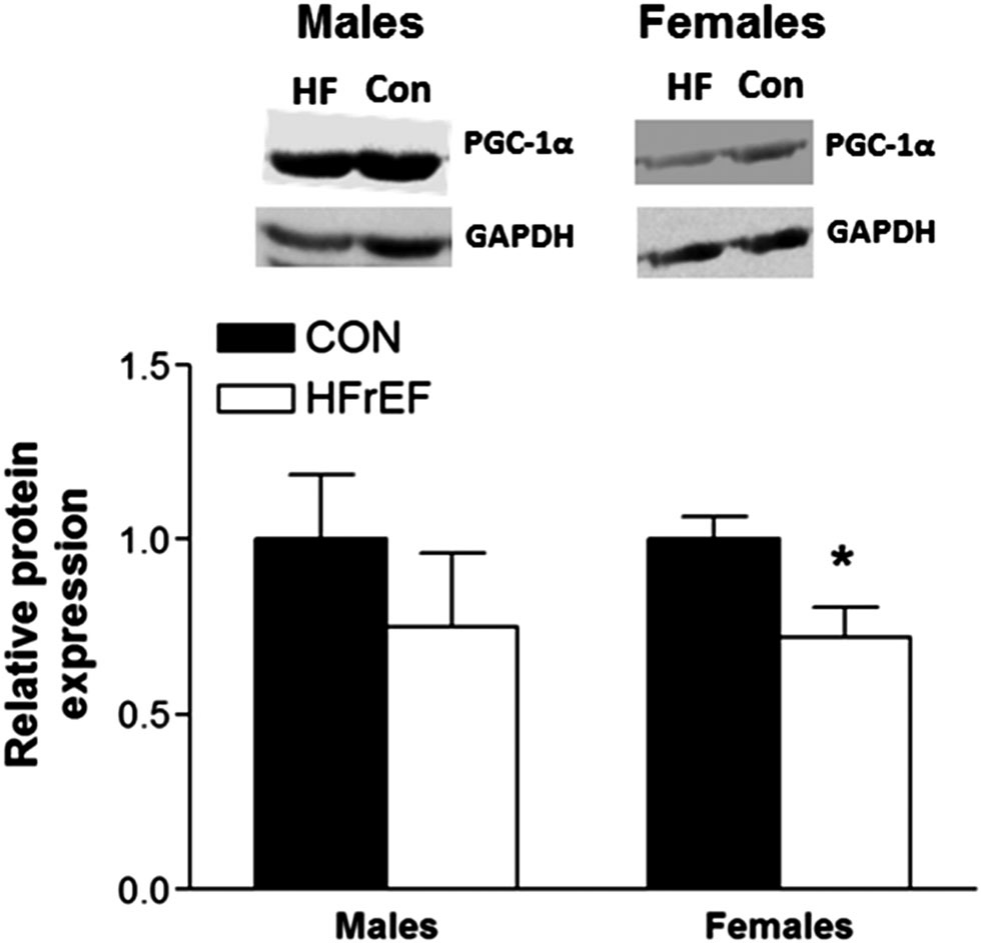
Relative protein expression levels of PGC‐1α. Compared with controls, female patients with HFrEF have lower relative protein expression while no difference was found in male patients (*n* = 8 per group for each sex). *P < 0.05 using unpaired Student's *t*‐tests. HFrEF, heart failure with reduced ejection fraction; PGC‐1α, peroxisome proliferator‐activated receptor γ coactivator‐1α.

Compared with female controls, women with HFrEF had a significantly lower relative mRNA expression in several key mitochondrial and respiratory complex genes involved in mitochondrial fusion, biogenesis, and oxidative stress (*Figure* 4). OPA1, a key fusion protein required for normal mitochondrial network integrity and dynamics, was 50.4% lower (*P* = 0.022; *Figure* 4A) in women with HFrEF. In contrast, no differences were observed in transcript levels of the key protein regulating mitochondrial fragmentation, FIS1 (*P* > 0.05; *Figure* 4B). The master regulator of mitochondrial biogenesis, PGC‐1α, was also lower in women with HFrEF by 71.7 % (*P* = 0.035; *Figure* 4C), while a similar trend was also found for the key mitochondrial‐anti‐oxidant SOD2, which was lower by 49.9% (*P* = 0.009; *Figure* 4D) in women with HFrEF. Finally, the expression of NDUFS1 and NDUFS3, both of which encode for core subunit proteins in complex I, were also 39.4% (*P* = 0.048; *Figure* 4E) and 32.2% (*P* = 0.034; *Figure* 4F) lower in women with HFrEF, respectively. In contrast, male patients with HFrEF did not differ in the gene expression measurements compared with controls (*Figure* 4A–4F) suggesting a systematic reduction in key mitochondrial gene expression specific to the skeletal muscle of female patients with HFrEF. We followed up these findings by confirming that protein expression of PGC‐1α was also lower in female patients (*Figure* 5; *P* = 0.022) but not in male patients (*Figure* 5; *P* = 0.398), when compared with controls.

### 3.4 Relationships between mitochondrial function, content, and gene transcripts

**Figure 6:**
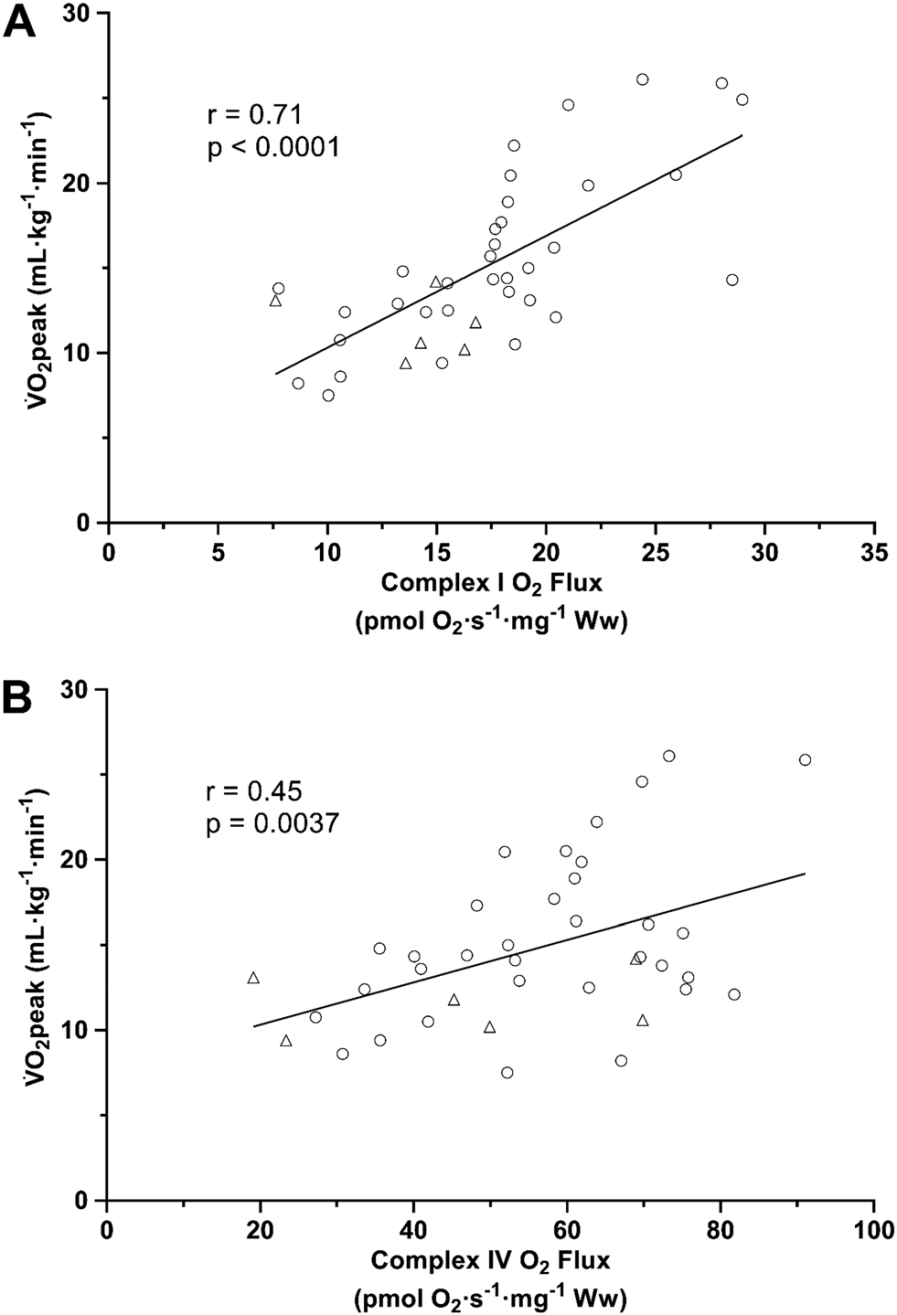
Measures of skeletal muscle mitochondrial function and content correlate with peak pulmonary oxygen uptake (V̇O_2peak_). Complex I O_2_ flux (A) and complex IV O_2_ flux (B) correlate with V̇O_2peak_ in HFrEF men (circles; *n* = 34) and women (triangles; *n* = 6).

Measures of mitochondrial respiration and mitochondrial content were compared against the V̇O_2peak_ measurements obtained from HFrEF patients during the exercise test (*Figure* 6). When comparing all HFrEF patients, there was a significant positive correlation between mitochondrial complex I respiration and V̇O_2peak_ (*Figure* 6A). Similarly, there was a significant positive correlation between mitochondrial complex IV respiration, as a marker of mitochondrial content, and V̇O_2peak_ (*Figure* 6B) across all patients with HFrEF. We also explored the relationships between the gene transcript expression levels and V̇O_2peak_ as well as mitochondrial function measurements in all HFrEF patients (*Table* 2). Of all the gene expression measurements, the only marker to show a trend for a correlation with V̇O_2peak_ was OPA1 (*Table* 2). OPA1, FIS1, NDUFS1, and NDUFS3 were all significantly correlated with complex I O_2_ flux (*Table* 2). Similarly, OPA1, PGC‐1α, SOD2, and NDUFS1 were all correlated with complex IV O_2_ flux (*Table* 2).

**Table 2:** Correlations between gene transcript expression levels with peak pulmonary oxygen uptake (V̇O_2peak_) and measures of mitochondrial function and content in heart failure with reduced ejection fraction patients

		OPA1	FIS1	PGC‐1α	SOD2	NDUFS1	NDUFS3
V̇O_2peak_	r	0.550	−0.118	0.043	−0.253	0.111	0.242
	p	0.080	0.729	0.900	0.453	0.746	0.501
Complex I O_2_ flux	r	0.642	0.616	0.448	0.293	0.646	0.584
	p	0.007*	0.011*	0.071	0.254	0.005*	0.017*
Complex IV O_2_ flux	r	0.498	0.436	0.572	0.534	0.490	0.384
	p	0.049*	0.092	0.016*	0.027*	0.046	0.142

FIS1, mitochondrial fission 1; OPA1, optic atrophy 1; NDUFS1, NADH:ubiquinone oxidoreductase core subunit S1; NDUFS3, NADH:ubiquinone oxidoreductase core subunit S3; PGC‐1α, peroxisome proliferator‐activated receptor γ coactivator‐1α; SOD2, superoxide dismutase 2.

* *P* < 0.05 using Pearson correlation.

## 4 Discussion

These data provide novel evidence that HFrEF induces divergent sex‐specific pathophysiological mitochondrial phenotypes in skeletal muscle that would be expected to predispose towards exercise intolerance, impacting mitochondrial ‘quantity' in women and mitochondrial ‘quality' in men. Major findings of the present study revealed that female patients with HFrEF demonstrated a lower mitochondrial content when compared with matched‐controls, which corresponded to a downregulation in the gene/protein expression of the key regulator of mitochondrial biogenesis PGC‐1α. In contrast, male patients with HFrEF presented with clear intrinsic mitochondrial dysfunction when compared with matched controls, with no alterations in mitochondrial content or aberrant gene/protein expression. Mitochondrial deficits closely correlated to V̇O_2peak_ across HFrEF patients, suggesting their clinical relevance in driving symptoms of exercise intolerance.

### 4.1 Impact of sex on skeletal muscle mitochondrial alterations in heart failure with reduced ejection fraction

A novel finding of the present study showed that, when compared with age‐matched controls, female HFrEF patients had lower mitochondrial complex I respiration (i.e. flux per wet muscle mass), but these differences were negated after adjusting for the significantly lower mitochondrial content (i.e. flux per mitochondrion). This finding therefore suggests that any differences in mitochondrial oxidative capacity in female HFrEF patients may be attributed to lower mitochondrial content rather than intrinsic mitochondrial dysfunction as observed in men. As such, female patients that have substantial muscle wasting (and by inference a reduction in mitochondrial content) would likely show the most severe impairments to exercise intolerance.^12^ Compared with age‐matched controls, previous studies measuring mitochondrial enzymes in patients with HFrEF have shown either no major changes^9^ or more pronounced deficits in men, the latter indicative of reduced content.^10^ However, these studies were concluded based on just a couple of oxidative enzyme measures/ limited mitochondrial functional data, inclusion of young patients (<60 years), and poorly matched cardiac function. In contrast to the female patients, we found that male patients with HFrEF exhibited a mitochondrial complex I‐specific impairment that was independent of mitochondrial content (i.e. an intrinsic qualitative deficit). This finding suggests that male and female patients with HFrEF have divergent responses to HFrEF in terms of skeletal muscle mitochondrial impairments. Thus, the current study significantly expands our understanding of the impact of sex on mitochondrial impairments in HFrEF. Furthermore, our data showed a significant association between complex I function and V̇O_2peak_ in patients, which firmly supports the evidence that mitochondrial dysfunction as an important mechanism of exercise intolerance in HFrEF.^21^, ^22^, ^23^ Future studies are therefore warranted on validating the current findings via non‐invasive or more accessible clinical approaches (e.g. near‐infrared spectroscopy^14^; Technetium‐99m sestamibi, ^99m^Tc‐MIBI and computed tomography,^24^, ^25^ or magnetic resonance spectroscopy^26^) in order to accommodate the wider CHF population (i.e. non‐pacemaker) and enable more targeted treatment of peripheral and/or central mechanisms limiting exercise intolerance in patients with CHF.

### 4.2 Mechanisms of sex‐specific mitochondrial alterations in heart failure with reduced ejection fraction

The expression levels of several key mitochondrial gene transcripts involved in mitochondrial shaping and maintenance (i.e. OPA1, PGC‐1α, SOD2, NDUFS1, and NDUFS3) were uniquely lower in female HFrEF patients, which corresponded to lower levels of mitochondrial content but not intrinsic dysfunction. These findings were not replicated in male patients with HFrEF, which clearly indicates sex‐specific divergences in potential molecular control mechanisms. In particular, we detected a significantly lower level of both gene and protein expression in the master regulator of mitochondrial biogenesis PGC‐1α. Given the key role PGC‐1α plays in maintaining mitochondrial content and exercise tolerance in disease,^12^ this finding could provide one mechanism for the sex‐specific differences we observed in terms of mitochondrial content between male and female CHF patients, and further why women present with worse exercise intolerance but respond better to exercise training compared with their male counter parts.^1^, ^2^ In addition, female CHF patients showed lower gene expression of OPA1 (a regulator of mitochondrial fusion) and SOD2 (a mitochondrial‐specific antioxidant enzyme), while FIS1 expression (a regulator of mitochondrial fission) was unchanged that suggests an equilibrium shift towards mitochondrial fragmentation and disruption of the mitochondrial network. Impaired OPA1 expression in skeletal muscle is known to impair mitochondrial morphology, induce apoptosis, and decrease mitochondrial content,^27^ while OPA1 is closely correlated to mitochondrial impairments and exercise intolerance in ageing.^11^ Further transcripts of specific respiratory complex I subunits (i.e. NDUFS1 and NDUFS3) were also downregulated in women with HFrEF, suggesting a widespread disruption of the mitochondrial signalling pathway is not induced within male patients. The finding that several genes were comparably lower in female patients with HFrEF would appear to suggest a coordinated downregulation in mitochondrial content, which we confirmed in further independent assays (i.e. complex IV and citrate synthase). That most of these mitochondrial gene transcripts were well correlated to mitochondrial respiration and content (*Table* 2) provide strong evidence these underpin, at least in part, the lower mitochondrial quantity and quality observed in patients with HFrEF. Interestingly, OPA1 also tended (*P* = 0.08) to be closely correlated to whole‐body V̇O_2peak_, supporting evidence that impairments to mitochondrial fusion may impair not only mitochondrial capacity but also overall skeletal muscle morphology, to impact exercise tolerance.^11^

The underlying mechanisms of why women respond differently in their mitochondrial morphology and function compared with male patients with HFrEF remains unclear but may be related to the sex hormone oestrogen or the preferential oxidation of fatty acids in women that may increase intrinsic function.^28^ The mechanism behind the lower intrinsic function of complex I in male patients with HFrEF remains unclear, but it may be related to a downregulation in complex activity/expression as transcriptional markers were not impacted.^29^ As such, future studies are warranted to help provide further mechanistic insight (e.g. signalling cascades) into the sex‐specific mitochondrial derangements induced by CHF.

### 4.3 Study limitations

This study was limited by the observational cross‐sectional design, which allowed characterization of variables and their relationships rather than prove causation and direct mechanistic insight. Therefore, an interventional study is warranted to address how skeletal muscle mitochondrial content and function change longitudinally in both male and female patients with HFrEF. When applying our findings to the wider CHF population, it should be considered that the data were collected from a moderate sample size where other confounding factors may play a role (e.g. cohort matching and physical activity levels). For example, we did not match participants for physical activity levels, which may have influenced our results.^30^, ^31^ However, detraining cannot fully explain mitochondrial deficits in HFrEF,^32^, ^33^ and we obtained samples from *pectoralis major*, which is less susceptible to the effects of detraining compared with locomotor muscles. Furthermore, we also did not have a measure of exercise intolerance (i.e. V̇O_2peak_) in controls to evaluate their current levels of aerobic fitness, while the assessment of muscle mass would have proved valuable at providing insight into the involvement of muscle atrophy into sex‐specific mitochondrial derangements.

## 5 Conclusions

This study presents novel evidence to indicate that male and female patients with HFrEF exhibit divergent mitochondrial responses in terms of content, intrinsic function, and gene/protein dynamics, which closely correlated with V̇O_2peak_. As such, these findings support the rationale for sex‐specific treatment therapies in HFrEF patients, with interventions targeting mitochondrial content in women and mitochondrial intrinsic function in men as an alternative approach to ameliorate symptoms of exercise intolerance.

## Conflict of Interest

None declared.
